# A Review on Fresh, Hardened, and Microstructural Properties of Fibre-Reinforced Geopolymer Concrete

**DOI:** 10.3390/polym15061484

**Published:** 2023-03-16

**Authors:** Prabu Baskar, Shalini Annadurai, Kaviya Sekar, Mayakrishnan Prabakaran

**Affiliations:** 1 Department of Civil Engineering, Sona College of Technology, Salem 636 005, India; 2 Department of Chemistry, Dongguk University, Seoul 04620, Republic of Korea

**Keywords:** alkali-activating solution, FRGPC, fibres, microstructural properties, mechanical characteristics

## Abstract

Alternative eco-friendly and sustainable construction methods are being developed to address growing infrastructure demands, which is a promising field of study. The development of substitute concrete binders is required to alleviate the environmental consequences of Portland cement. Geopolymers are very promising low-carbon, cement-free composite materials with superior mechanical and serviceability properties, compared to Ordinary Portland Cement (OPC) based construction materials. These quasi-brittle inorganic composites, which employ an “alkali activating solution” as a binder agent and industrial waste with greater alumina and silica content as its base material, can have their ductility enhanced by utilising the proper reinforcing elements, ideally “fibres”. By analysing prior investigations, this paper explains and shows that Fibre Reinforced Geopolymer Concrete (FRGPC) possesses excellent thermal stability, low weight, and decreased shrinking properties. Thus, it is strongly predicted that fibre-reinforced geopolymers will innovate quickly. This research also discusses the history of FRGPC and its fresh and hardened properties. Lightweight Geopolymer Concrete (GPC) absorption of moisture content and thermomechanical properties formed from Fly ash (FA), Sodium Hydroxide (NaOH), and Sodium Silicate (Na_2_SiO_3_) solutions, as well as fibres, are evaluated experimentally and discussed. Additionally, extending fibre measures become advantageous by enhancing the instance’s long-term shrinking performance. Compared to non-fibrous composites, adding more fibre to the composite often strengthens its mechanical properties. The outcome of this review study demonstrates the mechanical features of FRGPC, including density, compressive strength, split tensile strength, and flexural strength, as well as its microstructural properties.

## 1. Introduction

Changing creative and environmentally friendly works are required due to population growth and new global rules. In 2050, the worldwide production of concrete is expected to increase from 4.3–6.1 billion metric tonnes. For instance, China used a major % of the global concrete supply in 2019 [1]. To evaluate the high utilisation of ordinary Portland concrete, measurements of CO_2_ (carbon dioxide) outflow (about 7% of world carbon dioxide emissions) and an energy-burning cycle with typical necessary energy power of around 4.8 GJ/t are utilised (OPC) [2]. The second-highest central excise payer in India and a significant contributor to GDP is the cement industry (GDP). Along with the housing industry and infrastructure growth, India’s need for cement will undoubtedly increase. However, the cement industry makes heavy use of energy. Portland cement is an energy-based intensive process, requiring 4 GJ per tonne of energy, behind steel and aluminium production. With a production rate of 298 MTPA, India now has an installed capacity for cement of 500 MTPA. About 35% of the installed capacity of cement plants is found in the states of south India. India’s overall installed cement capacity is 325 MTPA, contributing to 65% coverage of the country’s total installed capacity under the Post Tensioning Association (PAT) programme. Due to the country’s developing infrastructure, India’s cement production was projected to reach five hundred million tonnes by 2020 and is now projected to reach eight hundred million tonnes by 2030. There are 130 big cement factories and more than 300 small cement plants in the cement industry. At the beginning of the 2008–2009 financial year, the sector’s capacity was around 198 million tonnes. In the long term, India’s demand for cement is predicted to increase by 10% yearly, driven by increases in capital spending for homes, infrastructure, and businesses. Given that the rise in production and consumption is predicted to be between 9 and 10%, the cement industry demand–supply balance is expected to improve over the long term, leading to significant CO_2_ emissions. Concrete made of eco-friendly geopolymers that emit less CO_2_ was discovered to solve this environmental problem.

Alkali-activated materials are one of the most promising materials for the substitution of Portland cement [3]. Geopolymers are alkali-activated binders, which are increasingly well liked as a low-impact alternative to OPC [4]. The current way of life, industry, and technological advancements have significantly increased waste quantity and variety in recent decades. The issue of waste disposal on both land and water results in several forms of environmental damage [5]. These waste by-products from industry and agriculture include FA, slag, RHA, bagasse, glass, tyres, etc. They are created by combining other source materials or Al_2_Sio_3_ sources, namely (FA), (VA), GGBS [6], and (MK). Due to greater SiO_2_ and Al_2_O_3_ concentrations and widespread availability, one of the current pozzolanic outcome materials is FA, which may be utilised as a binder material in concrete, which subsequently goes through the polycondensation process [7]. Numerous researchers have demonstrated that FA enhances mechanical capabilities, microstructural compositions, and durability [8,9,10,11,12]. The compressive strength of cement decreases when more than half of fly ash is substituted, according to prior research [13]. Nevertheless, geopolymers made solely from fly ash have acknowledged setting time and limitations as treatments. The end result is a product with high initial strength and maybe lower carbon dioxide emissions. Numerous factors, such as source material [3], activator solutions [14], and type of aggregates [15], affect geopolymer reactivity and its properties [16,17].

The initial features of the material (molecular size, aluminium, and silicon reactivity; Fe, Ca, and inert particle presence), the type and convergence of activator, and other reactive circumstances [18,19] are the most crucial ones. According to a stated assessment, geopolymer concrete functions at room temperature as a coupling framework, such as regular Portland concrete. If a geopolymer compound has to be heated to set, it could be better to refer to it as a geopolymer fastener rather than geopolymer concrete. Instead of standard Portland concrete, geopolymer concrete is a practical and innovative alternative for construction projects on land and at sea. It relies on mechanical processes or little processed natural resources to greatly reduce its carbon footprint. It is also resistant to many strength factors that might harm conventional cement. An alumina silicate substance, a simple antacid reagent (sodium or dissolvable potassium silicates), and water are required to make geopolymer concrete. The utilisation of GGBS increases concrete strength by lowering water absorption [20] and the use of 40% GGBS performed best due to the lowering of penetrable pores. Oil palm shell concrete (OPSC) with GGBS of up to 60% concrete replacement met the base stipulated strength required for lightweight main cement, despite high slag content being the cause of loss in strength (LWC) [21,22]. According to a study on green cement, waste materials and industrial by-products might be used with cement to increase mechanical and toughness properties. Depending on the application, they could also be used to replace all of the cement in a structure or only as a fibre support [23].

Fibres are often included in cement to avoid breakage brought on by drying shrinkage and plastic breakage. These fibres also make cement less permeable by obstructing water drainage. Fibre (steel) greatly impacts slag-based GPC’s mechanical properties, and the use of steel fibre decreases compressive strength while enhancing parting plasticity and bending capacities [24]. Slag that has been fibre-reinforced outperforms Portland cement concrete mechanically. Research on small steel fibres’ impact and fly-ash-based geopolymer concrete’s mechanical properties has shown that the expansion of small steel fibres enhances flexure and energy retention limit. The effects of single fibre and combination steel fibre in super-excellent conventional concrete [25] revealed that crossover steel fibre is more efficient at boosting cement’s capacity to transfer energy under effect load. Cement has been partially or entirely replaced by a range of industrial by-products in geo-polymer and conventional concrete [26,27,28]. It has been intriguing to see how different fibres, such as cellulose, sisal, jute, and bamboo, affect the brittle nature and inorganic matrix’s physical properties and mechanical properties. Cellulose fibre support greatly raises the fracture [29] and mechanical properties of epoxy gum [30,31]. Superior fibre-reinforced concrete has a substantial strain-hardening response, greatly increasing concrete’s strength. FRGPC is a frame cycle that uses a large amount of irregular fibres and drives a very thick plastic-like combination through an unbending aperture with optimal form. Extrusion was observed to produce concrete with greater flexural elasticity and strength compared to the conventional one [32].

Fibre may increase the framework’s flexibility, enabling them to successfully connect lattice microcracks. The length, measurement, arrangement, and conveyance of the fibres and grid features are where the danger element for the concrete occurs. Microcracks in the fibre that link them provide closing pressures that slow the growth of breaks. Because they are closer to one another for a given volume, microfibers may catch framework breaking at the microscopic level [33]. When a strong link between the fibre and the network is made, it is discovered that shortening the distance between well-distributed fibres improves concrete characteristics. Fibre length has an impact on concrete’s strength and durability. When considering the impact of fibre length, the total length should be taken into account. The initial capacity limit of fibre is built using this entire length as a base. Concrete with fibre lengths below the total length will not fracture because the fibre is not long enough to do it. This paper presents a review on the research works performed by various researchers on fibre-reinforced geopolymer concrete made with varieties of fibres, as well as different fresh, strength, and microstructural properties.

## 2. Geopolymerization

At temperatures lower than 100 °C, certain calcined clays, particularly calcined kaolinite (metakaolin), may be activated with an alkaline solution to form hardened ceramic-like products. Because of this finding, geopolymerisation has advanced. Several aluminosilicate materials, such as coal ash, metakaolin [34], calcined clay, agricultural waste ashes, and industrial sludge waste, can be used to make geopolymers. Silico-aluminates, which contain Si_4_^+^ and Al_3_^+^ in four-fold coordination with O+, are known as geopolymers. After lime concrete and Portland concrete, geopolymer is the third generation of concrete. In this application, “geopolymer” refers to alkali aluminosilicate, sometimes called inorganic polymers, salt-started cement, and geo cement [35]. It is composed of a sialate monomer reworking unit (Si-O-Al-O). The geopolymerisation cycle uses aluminosilicate minerals, such as kaolinite and feldspar, and contemporary strong industrial leftovers, such as fly ash, mining waste, and metallurgical slag as raw materials [36].

**Figure 1:**
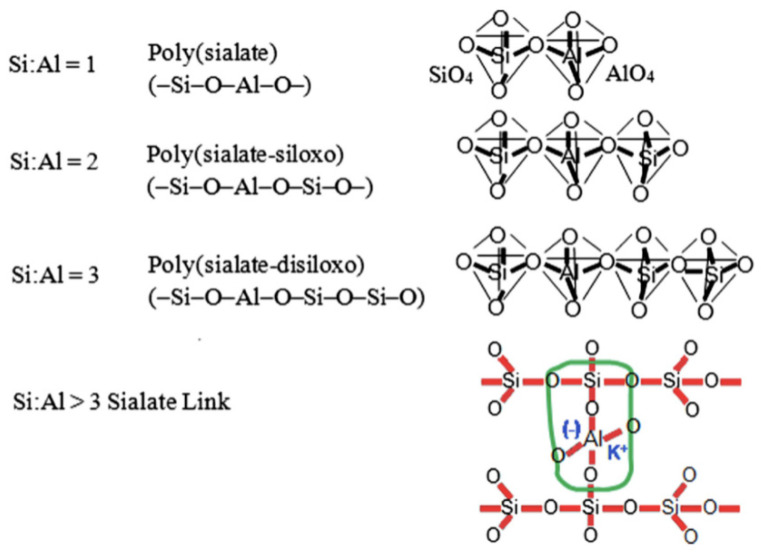
Chemical Reaction of Geopolymerisation [43].

The aluminosilicate source’s mineralogical makeup, morphology, fineness, and glassy content all impact how reactive they are [37]. To produce a stable geopolymer, the primary requirements are a raw material that is fundamentally formless; has sufficient responsive polished substance and less interest in water; and can transfer aluminium without difficulty. Aluminosilicate [37] materials are activated using essential activators, such as NaOH, KOH, Na_2_SiO_3_, and K_2_SiO_3_. KOH demonstrated a more pronounced alkalinity degree compared to NaOH. NaOH has shown a more remarkable capacity to liberate Si and Al monomers [38]. A study is currently being conducted to see whether potassium silicate may be replaced with less costly soluble volcanic materials to produce geopolymeric concrete. The potent Al_2_SiO_5_ powder reaction with an antacid hydroxide/soluble base silicate integrates geopolymers. A diagram shows how fly-ash-based geopolymers and concrete are arranged. When responsive aluminosilicates are broken down, and free [SiO_4_] and [AlO_4_] tetrahedral units are provided in arrangement, polymerisation occurs under very basic circumstances [39]. Tetrahedral units frame polymeric Si-O-Al-O bonds because they share an oxygen iota with the polymeric precursor. During geopolymerisation, the following reaction takes place [40]: Si(OH)_4_ + Al (OH)_4_- = (Si_2_O_5_.Al_2_O_2_)_n_ + H_2_O + OH. In this reaction, water is given, which normally burns through during disintegration and benefits the mixture throughout maintenance. This differs from how water reacts with Portland concrete during the hydration process, during which heat is created and water is absorbed [41]. Metakaolin/fly waste activation produces zeolite-type hydration outcomes. While C-S-H has a low calcium-to-silicate proportion, which is the important stage made during slag enactment, sodium aluminosilicate hydrate gels have varying silica-to-alumina proportions [42]. The chemical reaction of geopolymerisation is represented in Figure 1 [43].

**Figure 2:**
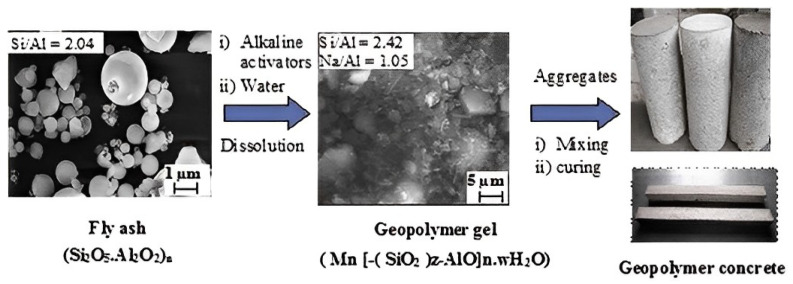
Stages of geopolymer formation [37]. (Reprinted with permission from the publisher Elsevier; License No. 5471710688327).

Even though many of the characteristics of geopolymers made from various aluminosilicate sources may seem to be the same, these polymers’ microstructures and material characteristics are very diverse [44,45]. They are normally created consistently with known properties throughout the design and progress. Metakaolin-based geopolymers have considerable leeway. However, because of the rheological problems caused by its plate-formed particles, the processing is more complicated, and the structure’s water interest is higher [44]. The geopolymer based on fly ash, however, is frequently more durable and grounded than the geopolymer based on metakaolin [38]. In comparison to metakaolin and fly-ash-based geopolymers, the slag-based geopolymer has been shown to have stronger early strength and more significant corrosive obstruction. Figure 2 shows the stages of geopolymer formation [37].

## 3. Fibre-Reinforced Concrete and Its Types

Concrete that has been reinforced with a fibrous material, or fibre-reinforced concrete (FRC), has higher structural stability. It has uniformly distributed, short discrete fibres that are randomly orientated. Steel, glass, synthetic, and natural fibres are all types of fibres. With variable concretes, fibre materials, geometries, distribution, orientation, and densities, fibre-reinforced concrete takes on distinct characteristics within these various fibres. Shotcrete uses fibre reinforcement the most, but regular concrete can also use it. In order to prevent plastic shrinkage cracking and drying shrinkage cracking, fibres are typically utilised in concrete. They also lessen the permeability of concrete, which lessens water bleeding through it. Greater impact, abrasion, and shatter resistance in concrete are produced by specific types of fibres [46,47,48]. The flexural strength of concrete is typically not increased by fibres, so moment-resisting or structural steel reinforcement cannot be substituted. The strength of concrete can be decreased by some fibres. Fibres assist in carrying the load by boosting the material’s tensile strength if their modulus of elasticity is higher than that of the matrix [49]. Flexural strength and matrix toughness are frequently divided when the aspect ratio of the fibre increases. However, too-long fibres sometimes “ball” up in the mixture and cause issues with workability. The impact resistance of concrete materials may not be significantly affected by the use of fibres in concrete, according to some recent studies.

The flexural strength and energy retention of geopolymer concrete have both been improved by the use of fibre in various forms, including steel, glass, hair, and nanoparticles. There are three essential criteria that must be taken into consideration when using fibre as a support in cementitious and geopolymer concretes as follows: (1) material qualities that are equivalent to those of the application; (2) fibre–framework cooperation to transfer stresses; and (3) appropriate perspective proportion to ensure strong post-breaking behaviour. Most importantly, the material’s schematic and mathematical characteristics of fibre are used in cement-containing material’s properties before looking at the concrete activity of fibres and geopolymers. Fibre-reinforced concrete may help in the resisting of more load [50,51] and reduce the formation or frequency of inward cracks in geopolymer concretes [52,53]. Fibre-reinforced concrete has better stiffness, flexural strength, and flexibility than conventional concrete-based materials. Their strength and energy absorption have significantly increased, and fibre in concrete helps to lessen penetrability in cases where strength has fractured. Substantial advancements have been made in geopolymer mortar, or solid, which was developed to replace the materials in current fibre-reinforced concrete [54]. It performs the role of a geopolymer fastener and results in better elasticity, a wider range of flexibility, and increased strength [55]. Although it is still in its early stages, fibre-reinforced geopolymer research is crucial.

### 3.1. Steel Fiber

Steel fibre is frequently utilised in cementitious concrete due to its high mechanical strength, flexibility, and availability. For specific uses, the American Standard’s Testing of Materials A820-16 lists five varieties of fibre i.e., steel, as follows: 1. twisted or smooth cold-drawn wire bits; 2. distorted or smooth sheets cut; 3. softened and separated; 4. factory cut; and 5. changed wire steel fibre. Due to the tiny size of these steel fibres, cement scatters them haphazardly [56,57]. Steel fibres may have a wide range of stiffness and extreme extensions between 0.5 and 3.5% and 310 to 2850 MPa, respectively, depending on the material kind and production measure [58,59]. According to the American Standard’s Testing of Materials A820-16, any of the ten samples’ base strength should not be less than 310 MPa, and its normal elasticity should not be less than 345 MPa. Metallic fibres have a folded surface because of their pliability and assembly techniques, which result in effective fibre–folio communication [60]. The main problem with steel fibre, notwithstanding a few interesting points of interest, is consumption [61,62]. Steel fibre is typically used in constructions that either (1) use treated-steel amalgams, preparations using hard materials, such as austenitic, ferritic, martensitic, duplex, and precipitation [63]; or (2) use-forfeited covering concrete, such as copper/zinc-covered prepares, to prevent corrosion.

### 3.2. Inorganic Fiber

The importance of inorganic fibres increases every day. Inorganic, as opposed to polymeric, fibres are made from substances found in, or are easily made from, substances naturally occurring in the earth’s crust. Glass, metals, carbon, asbestos, and ceramics are a few examples of materials that are now used or are being studied as sources of fibre. Asbestos is an example of an inorganic fibre that has been used since ancient times. These fibres are frequently employed in high-heat applications, such as refractories, because of their high melting points. They are composed of alumina and silica mixes. The key difficulties for these fibres include ease, high stiffness, material solidness, and wonderful protecting characteristics. Depending on the pre-existing material, various preparation techniques have been used to create inorganic fibres, including (1) bar drawing, which is used, for instance, in wire-making practices; (2) entry through an opening, which is used, for instance, in the creation of dissolve spun fibre; (3) fume statement, which is used for instance, to fume plate boron on a tungsten centre; and (4) gel formation from a soft arrangement or by the fume fluid strong strategy, for example, the amalgamation [64].

### 3.3. Glass Fiber

Glass-fibre-reinforced polymer composites have been created using various assembly techniques and are typically used for multiple purposes. At first, the ancient Egyptians made holders out of glass strands that were pulled from glass and heated up. The 1930s saw the invention of glass-fibre-infused processes for high-temperature electrical applications. These days, it is used in electronics, avionics, automobile applications, and other things. Each glass strand has unique qualities and is used in various polymer composite applications. Different glass-fibre-reinforced polymer composite’s mechanical, tribological, thermal, water assimilation, and vibrational characteristics were taken into consideration. Glass is typically created by melting silica, the substance that makes up sand, at extremely high temperatures, adding substances (such as oxides of various metals that give the glass the desired properties), and then forcing the molten glass through a spinneret. Glass comes in a wide variety of types and is used for a variety of purposes. Energy bills are always higher when the temperature is high. When metal oxides are removed from the ores in which they are contained in the ground, concerns should be raised regarding energy usage, the need for large extraction or refining equipment, and the creation of considerable amounts of pollutants.

### 3.4. Al_2_O_5_Si and Al_2_O_3_ Fiber

Al_2_O_5_Si fibres refer to several types of M_2_O_3_ fibres that comprise around 45 to 60% Al_2_O_3_; the remaining material is silicate. They are often conveyed by the blown or spun cycle of liquid kaolin or related muds or precursors, including aluminium oxide and silicon dioxide [65]. The Al_2_O_3_ to SiO_2_ ratio of these materials greatly influences their mechanical properties. If the alumina % is increased, fibre with 52% Al_2_O_3_ content may resist temperatures as high as 1250 °C, whereas a fibre with greater silica content has lower rigidity and a lower flexible modulus [66]. Additionally, the material’s fuzziness affects the fibre’s flexibility and heated shrinkage. For instance, the 1400 °C shrinkage and stiffness of an Al_2_O_3_ fibre that includes around 4% SiO_2_ are lowered from 18% to 0% and 1800 MPa to 500 MPa, respectively, when the crystal is enlarged from half to 100%.

### 3.5. Basalt Fiber

Basalt fibres are used as a substitute for glass because basalt is a rock created from volcanic lava that has hardened. Previously, they were only utilised as basalt “wool” for thermal insulation. They may be utilised in the temperature range of 200 to 800 °C and are more resistant to strong alkalis but are less resistant to strong acids than glass is [67,68]. It appears that the created filaments are uniform enough to be utilised in typical textile constructions. Sewing threads for materials exposed to hot temperatures or harsh chemical conditions is one application that has been suggested. As a result, the environmental cost of creating these fibres, which is mostly due to the high temperatures required for their manufacture, is somewhat compensated by their capacity to withstand heat, which grants longer existence in thermally deteriorating conditions. Basalt is a solid inert material abundant in strength, hardness, and warm qualities. Furthermore, it was shown that basalt fibre has strong resistance to corrosive attacks even if it degrades in acidic environments [69]. The material properties of basalt range from very low in temperature (about −200 °C) to very hot (700–800 °C). Higher temperatures may cause underlying changes in basalt [70].

### 3.6. Other Inorganic Fiber

Numerous inorganic fibres with high mechanical characteristics and warm obstruction were made for specific purposes, including boron, B_4_C, BN, zirconia, SiC, and Si_3_N_4_. Thought should be given to evaluating these fibres beyond this inquiry’s scope [53,71].

### 3.7. Plant-Inferred Fiber

Seeds (such as kapok, oil palm, cotton, and coir), bast (such as ramie, hemp, flax, jute, and kenaf), straw (such as wheat, rice [72], and corn), wood (such as soft and hardwood), grass (such as bamboo and bagasse), and leaves are also sources of plant fibres (e.g., pineapple, sisal, and abaca). They are essentially constituted of cellulose, hemicellulose, coconut fibre, banana fibre lignin, gelatine, waxes, and certain water-dissolvable components [73]; plant-inferred fibres are very much intended to be advanced concrete materials. One of the key characteristics of plant-based fibres is their greater capacity to absorb moisture, which is frequently a crucial need for manufactured composites. This ability is a result of the presence of a larger proportion of hydroxyl groups and their hygroscopic nature. Lumen and focal cavity, responsible for the plant’s water uptake, and a few divider layers, which are grouped in necessary and optional dividers, make up most of a single plant fibre’s structure [74]. To yield the growth of phones throughout the plant’s development, the primary divider (P) has cellulose micro fibrils that are oriented irregularly. Three sub-layers make up the auxiliary divider (S) [74]. Using helical winding as an example, the cellulose microfibrils of the additional divider layer show a specified direction. Cell dividers, implanted in a grid of hemicellulose and lignin, are composed of cellulose microfibrils coated with hemicellulose structures.

## 4. Mechanism and Matrix Interaction

The interactions between fibres and the brittle concrete matrix that result in physical and chemical adhesion, friction, and mechanical anchorage because of complex fibre geometry, deformations, or other treatments on the fibre surface are the primary factors that affect the mechanical behaviour of fibre-reinforced concrete (FRC). “First generation” steel fibres, which were created by shearing thin steel sheets, were ineffective because the matrix did not adhere to them well enough. The most crucial of the bonding methods, mechanical anchoring, was further improved using a variety of fibre shapes. Similar surface modifications have been used on synthetic fibres (mainly polypropylene) to strengthen the connection between the fibres and the matrix [75]. When FRC is strained, there is initially an elastic stress transfer between the fibres and the matrix (either by external loads, shrinkage, or thermal stressors). Because the elastic moduli of the matrix and the fibres are so different, shear stresses develop at the fibre/matrix interface. After shear stress at the contact is exceeded and frictional shear stresses take over as the primary stress transmission mechanism, debonding gradually occurs. At some point during this gradual transition from elastic to frictional load transfer, the matrix starts to crack, and the debonded sections start to move.

The bending moment’s tensile stress changes into shear stress at the fibre–matrix interface in a fibre-reinforced composite, which is resisted by adhesion and friction at the surfaces where they make contact. Both at the interfaces of the two components and farther away from the interface, this contact force affects the geopolymer matrix. As a result, the matrix and fibres work in tandem to form an annular zone that encircles the fibres [55,76]. However, this force is greatest when the two components converge and dissipate as one retracts from the surface until it is zero [55]. This mechanism is comparable to that utilised in steel-bar-reinforced concrete, where the ribs that form on the bar’s surface spread the applied stress and raise the section’s load capacity [77]. When there is significant interfacial contact between the fibre and the matrix, such as when steel fibre is employed, all four processes take place.

**Figure 3:**
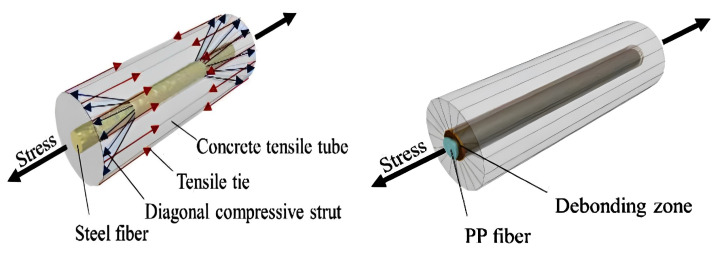
Matrix Interaction [85]. (Reprinted with permission from the publisher, Elsevier; License No. 547170968094).

Contrarily, for hydrophobic fibres with minimal roughness, the transfer of stress between the fibre and matrix is reduced, and each operates independently [78]. Numerous studies [79,80] have shown the capability of surface modification to increase the adherence of fibres with hydrophobic properties, such as polypropylene (PP) and polyester (PET) fibres. Several techniques, such as chemical surface modification (such as alkaline and silane treatment) [81,82], mechanical modification (such as fibrillation and micro-indentation) [83], and plasma modification, have been used for this purpose. These techniques’ key concepts are activating the fibres polar groups and increasing surface roughness. However, they sometimes negatively impact fibres by reducing their cross-section, which affects the composite’s mechanical characteristics. Additionally, the hydrophilic fibre’s surface must be coated with oil for specific applications, such as when the strain-hardening behaviour of geopolymer composites is important, to minimise adhesion and lessen the fibre–binder interaction [84]. Figure 3 provides information on matrix interactions [85].

## 5. Materials

### 5.1. Fine Aggregate

As a fine aggregate, river sand from a nearby source is employed in many studies. As partial substitutes for fine aggregates, a variety of industrial waste products [86], including quarry dust, glass powder, ceramic dust, and coal dust, can be used. After replacing with by-products, strength standards are assessed, as well as profit percentages. After this, the strength difference is not significantly bigger; however, taking into account cost, it can replace up to 40% of the quarry dust to generate a profit of about 10%. Like glass powder, ceramic dust can only be replaced by 20% without reducing strength. Even with a 20% replacement, it can still make 1.34% and 2.42% of the profit. Because it weakens the material, coal dust is not a good substitute for fine aggregates.

### 5.2. Coarse Aggregate

In stone quarries, coarse aggregates are often produced by blasting, breaking by hand, or using crushers. Stones that have been crushed by a machine come in several sizes, but aggregates that have been broken by hand only come in one size. To make graded aggregates for premium concrete, they are mixed once more in prescribed ratios. In order to utilise them in concrete, they must be well cleaned. Locally available coarse aggregates are used in many studies. Actual aggregate qualities were examined by method 49, supported by BIS 2386-1963. The findings should satisfy that the characteristics of totals meet the specifications recommended by BIS 383-1970. In structural concrete, coarse aggregates are employed as broken pieces of hard rock, such as granite and limestone (angular aggregates) or river gravels (rounded aggregates). For non-structural mass concrete of low strength, coarse particles such as cracked bricks, foamed slag, clinker, etc., may also be used. Coarse aggregates, including coal, lignite, soft bits, and clay lumps, should not include more than 5% foreign materials by weight. Aggregate size restrictions for different types of non-reinforced construction should range from 40 to 75 mm (1.5 to 3 inches).

### 5.3. GGBS

According to [87], when compared to other fillers, GGBFS is off-white and thus produces excellent cement concrete. GGBFS is made from iron, a by-product of blast furnaces. When limestone and iron metal coke melt at 1500 °C in a heater, molten slag and liquid iron are produced. However, after being heated and dried, slag is passed through a ball-plant to create GGBFS, a very fine powder. It has high silicate content and alumina content [88]. GGBFS is extensively used and replaces Portland concrete by around 70% [89]. GGBS has a 2.91 specific gravity and 3.11 fineness.

### 5.4. Metakaolin

Clay kaolin is used to make metakaolin, which is then lit under carefully controlled circumstances to produce an aluminosilicate with no shape. The cleaned kaolin is placed in a revolving oven, compressed into a fine powder, and then stored to create metakaolin. When kaolinitic clay is calcined at a temperature ranging from 600 to 700 degrees Celsius, metakaolin is produced. Metakaolin is a very reactive and delicate substance. Thus, it provides a rich, non-tacky surface to make the delivery of final concrete easier. It may be used as a solid admixture and is cured similarly to regular concrete. You may use metakaolin as a partial or complete additional cementitious ingredient.

## 6. Alkaline Activator Solution

Table 1 explains the chemical properties of various materials, and Table 2 shows the physical properties of various materials. NaOH and Na_2_SiO_3_ solutions both are components of an alkaline liquid. Sodium silicate available in liquid form. NaOH is 97–98% pure and comes in flakes or pellets. These flakes or pellets might be dissolved in water to create a NaOH solution. The mass of NaOH solids in a solution is impacted by its molar or M concentration. The molecular weight of NaOH is 40. The solution prepared using flask with a capacity of 1 L, and a 1 L solution is made by gently adding NaOH flakes to distilled water. For instance, 320 g of NaOH flakes are weighed and may be dissolved in 1 L of distilled water to create an 8 M NaOH solution. To make fresh concrete more workable, a high-range, water-reducing, naphthalene-based super plasticiser can be used. More water and roughly 15% of the binder are added to make the concrete more workable.

## 7. Specimen Preparation and Testing

Fly ash, FA, CA, Na_2_SiO_3_ arrangement, NaoH arrangement, and fibre arrangement were used to lay out test instances. All of the sums were set up in a dry, surface-soaked condition. Before being added to the dry components, sodium hydroxide and sodium silicate arrangements were mixed over the course of one day. Dry materials were first blended in a blender of the drum type with a 1.5 concrete-filled tube (cft) (0.062 m^3^) limit. After being mixed with the soluble arrangement, the super plasticiser was applied to the dry materials. Steel fibres were introduced in the appropriate quantities throughout the mixing process. Freshly blended fibre-reinforced geopolymer concrete (FRGPC) was poured layer-by-layer into standard 3D shapes with dimensions of 150 × 150 × 150 mm to test concrete’s compressive strength; 150 mm and 300 mm cylinders for parting tensile testing and Poisson’s proportion; and into crystals with dimensions of 100 × 100 × 500 mm to find the modulus of rupture [98]. There were three stages altogether. On a vibrating table, each layer was vibrated for 15 s. After compaction, a smooth scoop was used to level the top surface. Moisture evaporation stopped, and plastic sheets were then placed on top of the moulds. Before moving to the steam repair room, the covered example rested for three days. A temperature of 60 °C was used for the restoration process for 24 h. Accordingly, examples of fibre-reinforced concrete and regular concrete (UHPC) were tested 7, 14, and 28 days after curing [99]. In particular, similar casting and testing techniques were utilised. The modulus of elasticity was determined using simple testing techniques.

## 8. Mixing Process

Table 3 describes the mix ratio. For regular cementitious and artistic concrete, 3D printing, slip framing (moving-structure expulsion), expulsion (through a fixed bite of the dust), slurry penetration, sheet creation with vacuum or without vacuum or pressing factor shaping, cold and hot squeezed sintering, and various blending and setting advancements have been used to create geopolymers. These many techniques yield production of various geopolymer concretes using almost every fibre, formula, and material.

**Figure 4:**
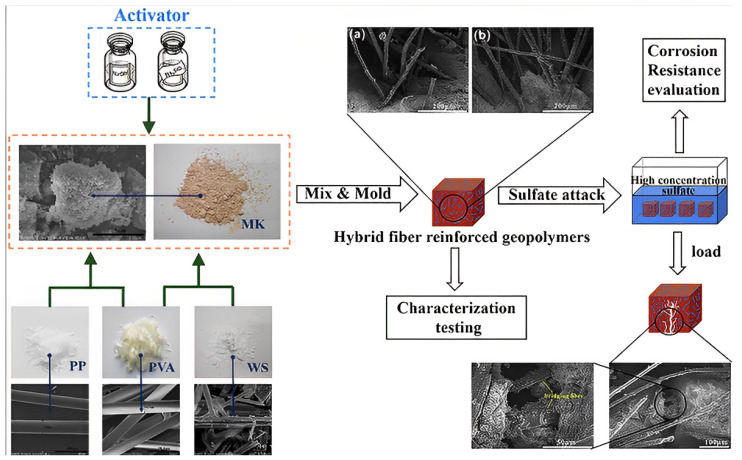
Mixing Stages of Geopolymer Concrete [98]. (Reprinted with permission from the publisher, Elsevier; License No. 547171104179).

In any event, uniform fibre dispersion requires a request for fibre consolidation during mixing. Before exposing them to a salt activator, progressively mixing the dry precursors with monofibres (such as steel and carbon fibre) has often been suggested [100,101,102]. Meanwhile, multifibres do not split and scatter evenly during the dry blending step (for example, multifibre PP fibre). Fibres should be mix after adding the solutions [76]. Dry alumino silicates and other fillers are then added. This improves the fibre’s wetting cycle, which raises fibre–grid communication. Comparative methods have been used to study how nanofibres and supports in geopolymers disperse. Before being added to the geopolymer network, graphene nano platelets were ultrasonically separated from other water particles [103,104,105]. When using a weak fibre, it is crucial that shear power—the force used to squeeze fibres into the geopolymer grid—be considered. The subject of this line of investigation is the basalt fibre breaking during blending when a shear blender or an outward blender is utilised. In close proximity to these techniques, which are often employed to mix short fibres, a high slurry geopolymer lattice and vacuum framework have been used to successfully impregnate the fastener through the blocked consistent fibre or texture fortifications. When the folio barely penetrates fibre organisation, unwanted porosity may be seen. Using alumino silicate particles with more than one fibre measurement component removes this porosity. Figure 4 describes the mixing stages of geopolymer concrete [98]. The SEM images shown in Figure 4 depicts how the fibres bridging the effects in concrete. 

**Table 3:** Mix ratio.

Ref	Composition	GGBFS/CFR/m^3^	FA	Metakaolin, kg/m^3^	Fine Aggregate, kg/m^3^	CA kg/m^3^	NaOH kg/m^3^	Cement kg/m^3^	Na_2_So_4_ + NaOH kg/m^3^	SiO_2_ kg/m^3^	Na_2_Sio_3_ kg/m^3^	Fibre kg/m^3^	M	SP %	H_2_O
[87]	Liquid ratio: 0.5b	143.32–592.41	-	146.84–572.3	1705	-	89.45–116.5	-	-	190	-	-	-	-	-
	GGBFS/CFR-M50	143.32–592.41	-	146.84–572.31	1705	-	89.45–116.5	-	-	190	-	-	-	-	-
[90]	SF0.5 NS2	225–22.8	225–22.8	-	859.69–865.22	737 742.88	-	450	225	-	-	0–10 (NS) 0.784 (SF)	12	5	8
[91]		-	6000	-	1430	780	10	-	-	-	1500	0.5% (PF)	-	-	-
[89]	F3 AR_80_ V_0.75_	39.4	354.95	-	554.4	1294	45.1	-	-	-	112.6	19.63–58.88 (SF)	-	11.8	59.1
[92]		-	639	-	285	975	72	-	-	-	180	-	-	-	53
[105]	PVA1.0 + Steel 1.5	-	-	-	1	-	-	0.5–1	-	0.05	-	0.5–2.5 (SF)	-	-	0.45
[106]		-	400	-	660	1230	-	400	-	-	2.5	-	12	2	14
[106]		SLAG 78–310	388–698	-	-	-	-	-	-	0–78	-	0–2 (SF)	-	-	-
[107]		-	400	-	540	1260	57.1	-	-	-	143	0–1.25 (Glass)	-	-	-

## 9. Results and Discussion

### 9.1. Compressive Strength of FRGC

Specimens with nanosilica (NS) have lower compressive strength than those without NS, which is relevant [90]. This outcome might be a result of unreacted NS, which severely dehydrates [108] the mixtures, causes fractures, and causes compressive strength to decrease. The compressive strength of self-compacting geopolymer concrete (SCGC) specimens is, however, marginally improved by the addition of steel fibre [109]. The compressive strength of plain geopolymer mortar for all mixtures during the early and late curing periods was enhanced by higher GGBS content, according to the results of [110]. This increase in strength is brought about by the combination’s increased calcium concentration, which creates a compacted microstructure. As a result of their larger size and lower pozzolanic activity, the agglomerated particles of densified silica fume (DSF) have a more porous microstructure and less strength. With the exception of samples with very low GGBS% (10% GGBS), the compressive strength of steel-fibre-reinforced geopolymer composites with integrated silica fume was equal to or greater than the plain geopolymer when taking into account the effect of silica fume form. However, 20% GGBS combination without silica fume reduces compressive strength by 8%. GGBFS/CFR-M25 showed a maximum strength of 12.75 MPa at the age of one day, but GGBFS/CFR-M100 generated a minimum strength of 3.21 MPa [84]. However, after 28 days, a GGBFS/CFR-M100 mortar mix’s compression strength dramatically increased.

GGBFS/CFR-M25 mortar mix has not achieved desired strength at the age of 28 days. Compressive strength improves initially before decreasing as NaOH concentration increases from 5% to 20%; the turning point was discovered at 10% [91]. Higher NaOH levels cause alumino silicate gel to precipitate, reducing compressive strength due to excessive hydroxide ion concentration. NaOH can therefore be used to boost compressive strength rather than thermal insulation. The compressive strength of specimens containing flax fibres indicates, in accordance with [111], that the injection strategy generates stronger samples than the traditional method. Specimens with fibres had 6% higher compressive strength than specimens without fibres. It should be noted that the short flax fibres added into the geopolymer had no effect on compressive strength due to the resistance fracture under axial stress. Compressive strength increases at all ages when hook-end steel fibres are added, as demonstrated by a specimen with 10% GGBS and 90% FA mix. The hook-end steel fibre’s greater aspect ratio raises the compressive strength of the GPC.

**Figure 5:**
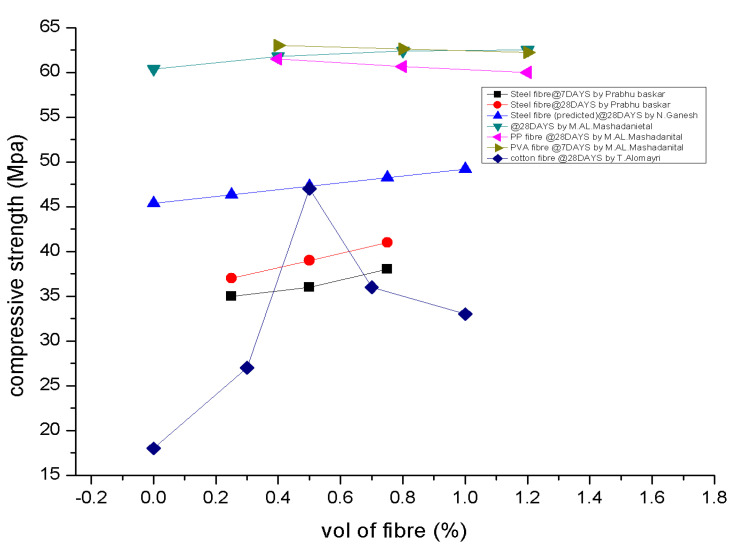
Compressive Strength [18,102,113].

Comparing the F3-AR80V0.75 concrete specimen to the GC specimen, the compressive strength improves by 33%. It should be mentioned, according to [92], that adding fibres to GPC does not significantly boost its compressive strength. The development of the compressive strength of steel-fibre-reinforced geopolymer concrete (SFRGPC) with different fibre volume fractions. The initial fracture was influenced considerably by the concrete’s compressive strength, which rose together with initial cracking stress [93]. Glass-fibre-reinforced polymer-reinforced ordinary Portland concrete (GFRP- ROPC) beams produce larger fracture width values than GFRP-RGPC beams, and bigger crack width values are reported for beams with low compressive strength. The composite with the highest compressive strength contains 0.5% cotton fibres. A balanced distribution of cotton fibres throughout the matrix, which improves the interaction between the fibre and the matrix, might lead to advantageous behaviour. The compressive strength of geopolymer composites containing between 0.7 and 1% by weight of the cotton fibre is inadequate. Poly (Siloxo-Sialate) (PSS)-added concrete has greater strengths than plain cement concrete [112]. With 1% basalt fibres added, compressive strength dropped by 26.4% and 12%. Figure 5 depicts the compressive strength [18,102,113].

### 9.2. Splitting Tensile and Flexural Strength

Splitting tensile strengths for PC concrete were reduced by 26.4% and 12% with the inclusion of 1.0% basalt fibres. Compared to PSS concrete without fibres, all PSS concretes with fibres showed increases in splitting tensile strength. Even for modest fibre volume contents, the composite peak stress levels may be 25–30% greater than matrix tensile strengths [112]. A brittle matrix may absorb more energy and become more ductile and resistant to fracture formation when ductile fibres are added to it. Tensile strength is only slightly improved.

**Figure 6:**
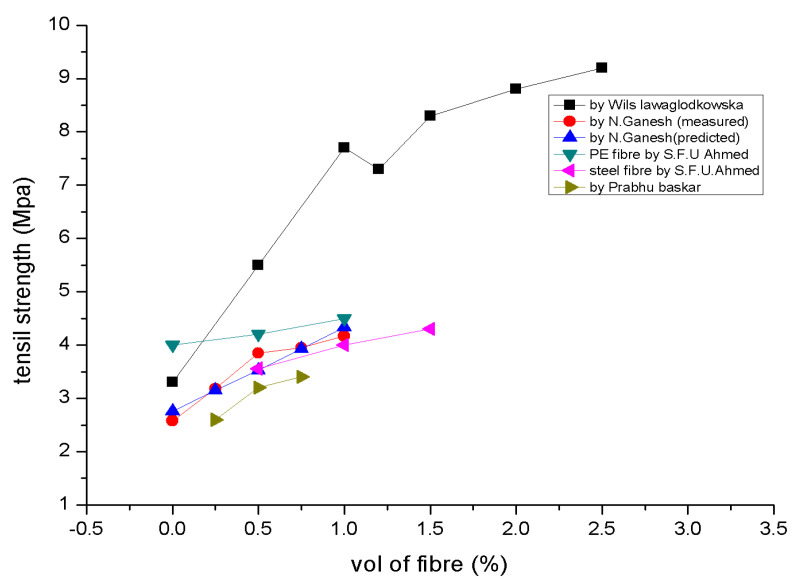
Splitting Tensile Strengths [89,92,105].

After reaching its maximum strength, gradual strain softening takes place, with the softening stage governed by the presence of fibres, much like its tensile behaviour [114]. Figure 6 evaluates the tensile strength [89,92,105,115].

**Figure 7:**
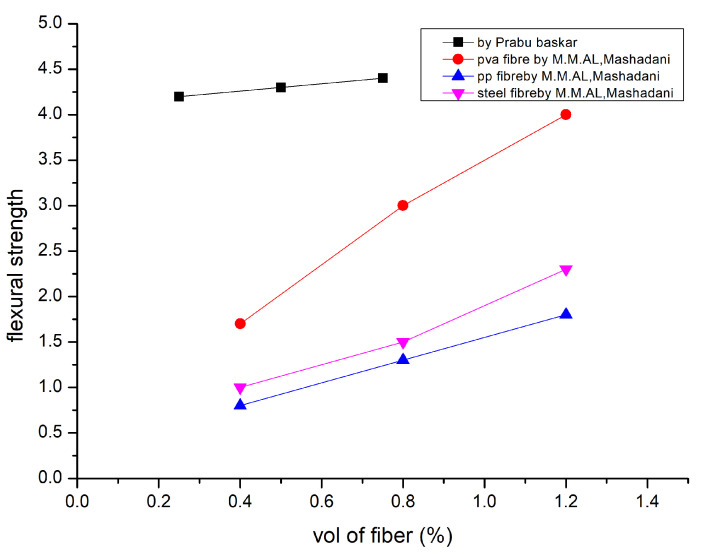
Flexural Strength [10,89,115].

Comparing ultra-high-performance fibre-reinforced concrete (UHPFRC) to UHPC, steel fibre content significantly improved tensile strength. It can be demonstrated that when compressive strength rises, splitting tensile strength climbs [116]. The A1 mixture, A3 mixture, and A5 mixture all had split tensile strengths of 2.28, 2.07, and 1.64 MPa, respectively, after 28 days. Regarding completely substituted cement with GGBFS, as well as added steel and glass fibres, these hybrid fibres exhibit an enhanced split tensile test. The findings imply that the inclusion of polypropylene (PP) fibres have an impact on fly ash concrete’s splitting tensile strength. At 28 days, concrete containing 60 and 120 kg/m^3^ fly ash had splitting tensile strengths similar to that of PC concrete. The optimal fibre content for all concrete is 0.45 kg/m^3^, according to the splitting tensile strengths of the concrete mixture. According to [117], strength ratings for engineered cementitious composite (ECC) without slag were much lower than those for slag tensile ECC (5.2 MPa) [118]. Hardened concrete properties are explained in Table 4 [16,44,56,95]. Figure 7 represents flexural strength [10,89,115].

### 9.3. Impact Strength

According to [95], composite’s low-impact strength reduces when fibre concentration rises to more than 0.5 weight percent. Cotton fibre concentration grows as the system’s viscosity increases, resulting in the formation of fibre agglomerates and voids, which reduce the adherence of the fibre matrix. The impact strength decreases as a result. Fibre reinforcing improves dynamic properties under impact and blast loadings, in addition to static energy absorption. It is important to note that different experimental methodologies have been used in the few studies that have looked at the dynamic properties of geopolymers reinforced with fibres. As a consequence, it is impossible to compare the findings directly. The split Hopkinson pressure bar (SHPB) apparatus, with a diameter of 100 mm, was utilised in a basalt-fibre-reinforced geopolymer concrete experiment [119]. The results reveal that the mechanical properties of the binder and loading strain rate have a substantial impact on the impact characteristics of geopolymer composites [119]. It was demonstrated that composites with higher strength have less strain rate sensitivity. In addition, the composites with stronger binders have a lower deformation capacity, despite having higher energy absorption. The addition of the fibre improves this drop in deformation capacity without affecting dynamic compressive strength [120]. An investigation employing the ITR-two hundred RADMANA impact test based on high-pressure gas punching on polyvinyl alcohol FRGC found that the addition of fibre transforms the brittle impact failure mode of geopolymers into a ductile one [121]. A new testing method was utilised to evaluate a steel-fibre-reinforced geopolymer by counting the number of 4.75 kg drops that were inserted into the centre of specimens, which were 400 mm in diameter and 40 mm thick, until they finally collapsed. The impact hardness of the geopolymer composite improved by 1%, from 233 to 4124 Nm [122]. The impact resistance of geopolymer composites was also tested in a fresh experiment employing a rectangular geopolymer bar with cotton fibre reinforcement that had not been notched, a Zwick Charpy impact machine, and a one J pendulum hammer. The impact strength of the geopolymer matrix was found to be doubled by adding 0.5 % of fibre. Fibre addition has a detrimental influence on impact strength [95]. The reason for this undesirable result is poor fibre dispersion inside the matrix.

### 9.4. Modulus of Elasticity

**Figure 8:**
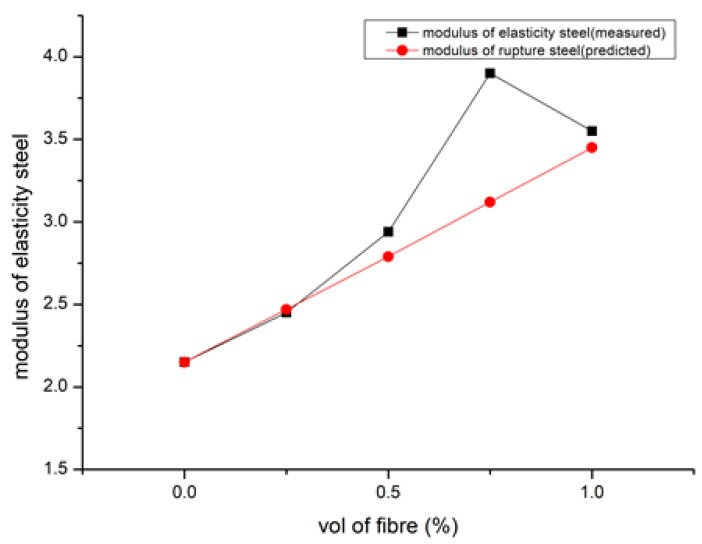
Modulus of Elasticity in MPa [92].

According to [92], SFRGPC’s elastic modulus values neared that of observed ones because inaccuracy was less than 0.002%. Figure 8 shows the modulus of elasticity [90]. GPC’s coefficient, 3190, is much lesser than the OPC value, 5000, which is stated in IS 456: 2000. This could be explained by the fact that GPC mixes reduce volume of aggregate, as observed by [105]. Their work [105] asserts that due to their low modulus, PE fibre composites have low peak tensile strength and high peak strain capacity, whereas steel fibre composites have high peak tensile strength and low peak strain capacity. To create a composite [33], basalt fibre was chopped and combined with clay and polypropylene. This method significantly increased the composite’s elastic modulus and yield strength. Hardened concrete properties are shown in Table 4 [92,116,123,124].

**Table 4:** Hardened concrete properties.

Fibre Used	Fibre %	Compressive Strength	Tensile Strength	Modulus of Elasticity	Ref.
SF	-	35–42.50	3.15–4.90	26,678.23–41,025.36	[92]
SF	-	20.21–40.63	1.55–3.01	2.94–11.39	[116]
SF	0–1%	26.9–90	-	3660–6137	[123]
PP	-	59.5–78	5.7–7.9	-	[124]

### 9.5. Workability

**Figure 9:**
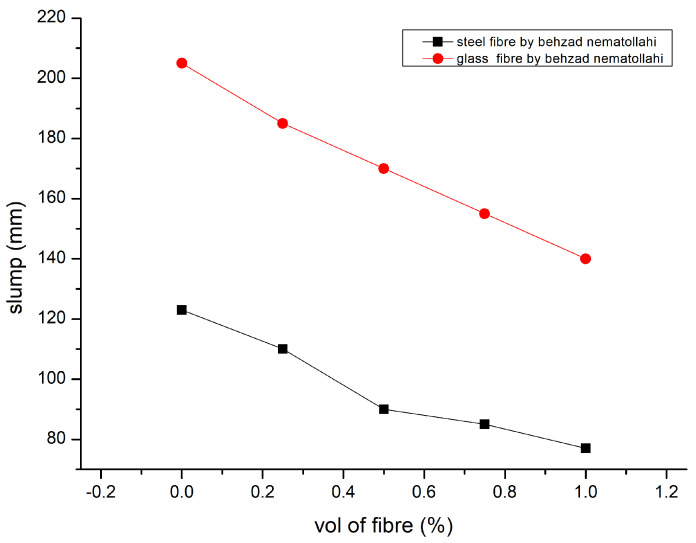
Slump Result [84,107].

**Figure 10:**
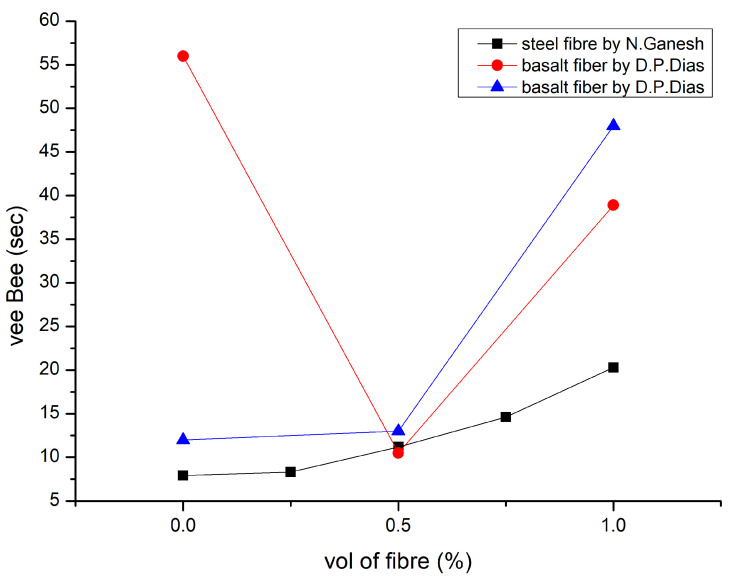
Vee Bee Results [92,112].

According to the analysis presented in [90] of the results from the most recent state tests, combining steel fibre with nanosilica had a detrimental effect on the droop flow diameter, flow time, V-funnel flow time, and L-Box passing ability. Mixes that included the most nanosilica (NS) (2%) and silica fume (2%) exhibited the biggest drops in fresh performance (1%). The self-compacting geopolymer mixtures fulfilled TS 12350 standards and EFNARC criteria for flow ability and passing ability. Figure 9 describes the slump result [84,107]. The volume proportion of fibres increased, and the workability significantly decreased [92]. The super plasticiser dosage was altered to preserve slump values. The Vee Bee time technique may also be used to evaluate GPC mixes, which are highly stiff and more workable during vibration. Figure 10 explains the Vee Bee results [92,112].

Silica fume affects the flow characteristics of FA and slag-based geopolymer mortar in a number of different ways [106]. Silica in a slurry and an undensified-form set worked significantly more quickly. This is explained by the extremely tiny silica particles that quickly combine with the alkaline solution and generate a gel with great capacity to hold onto water. Densified silica fume was added, but it made no appreciable difference in workability. Fibres reduce the workability of cementitious composites despite their superior mechanical properties, which leads to excessive void formation and insufficient compaction. Therefore, the application requires a balance between new traits and hardened behaviour.

**Figure 11:**
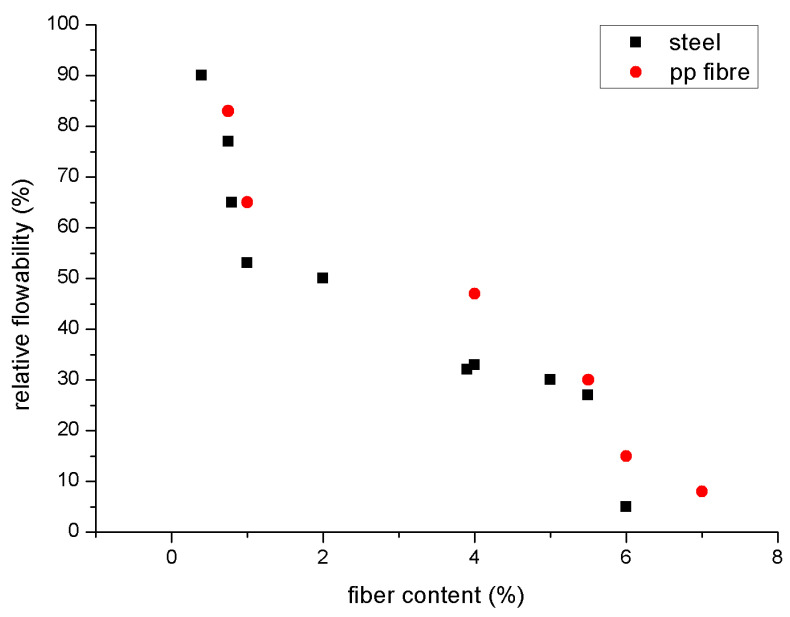
Relative flowability [76].

**Figure 12:**
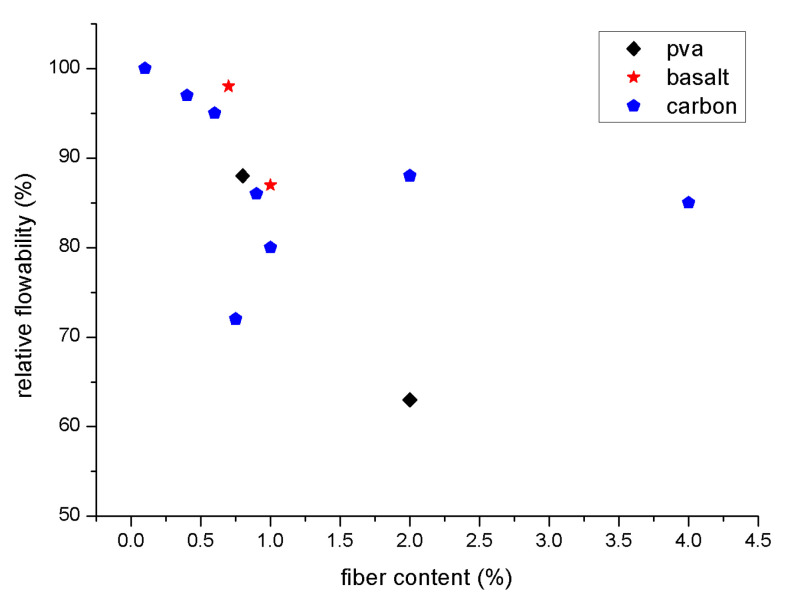
Relative flowability [85].

Figure 11 and Figure 12 explain relative flowability [85]. No matter the kind or structure of the fibres, the flowability of geopolymer composites reinforced with fibres decreases as fibre content rises. Standard flowability and slump tests (ASTM C143 and C230) may be reduced; the Vee Bee time test and time passing through a V-funnel may be increased [112,125]; the time passing through a V-funnel may be decreased; or the increase in yield stress and the increase in fibre content, equivalent diameter, and aspect ratio may increase the contact network between stiff fibres. It is suggested that the essential concentration value for geopolymer and cementitious composites should range from 0.2% to 2% [113,126]. When fibre content surpasses a particular concentration, even extremely flowable matrices may not travel through the congested fibre network efficiently because the fibres have a tendency to form clumps or balls and generate uneven dispersion. In order to overcome its severe static mode and manufacture the mould, the new matrix requires extra vibration [85].

Numerous studies have demonstrated that the crucial value often declines as the fibre and filler content aspect ratio increases [127,128,129,130]. Increased surface roughness and crimped or hook-end fibres also result in a reduction in critical concentration and flowability [113,131]. However, the distribution and orientation of fibres, as well as the mechanical characteristics of the whole composite, are influenced by plastic viscosity, yield stress, and flow confinement conditions of the fresh composite. Due to the greater yield stress in the new matrix, compression strength would fall for the same fibre content. A well-ordered fibre alignment may be accomplished for matrices with a low yield stress, directly improving the flexural behaviour of the composite [32,130]. The decrease in workability brought on by the presence of fibres has been constructively employed to increase the thixotropy of the new matrix, which improves the build ability and forms the stability of fibre-reinforced geopolymer composites for concrete extrusion and 3D printing [132,133].

### 9.6. Shrinkage

Furthermore, polypropylene and polyvinyl alcohol fibres significantly reduce drying shrinkage due to the same fibre type’s reducing shrinking scale and rising fibre dose. The shrinkage values for steel-reinforced composites fell from three 35 to two 81 micro strain on the 84th day of drying. According to [134], these composites’ thermal shrinkage studies reveal that, despite the fact that microcracks can occur up to 400 °C, fibres are thermally protected by the matrix, opening up new possibilities for inexpensive, quickly manufactured construction materials. Significant capillary pressure that develops between the micropore network’s wet and dry zones is the cause of the drying shrinkage of geopolymers, which causes specimen deformation and crack initiation [135]. Capillary porosity must be reduced to limit water loss during curing. The following two techniques have often been used: (1) changing the pore structure; and (2) adding reactive or inert fillers and fibres [136]. Numerous investigations have shown that the quantity, modulus, and interaction of fibres with the binder are the main factors that influence how much reinforced geopolymer composites shrink. The addition of two fibres in reduces the shrinkage in fly ash based geopolymer. The drying shrinkage of the composite specimens was significantly reduced, even with a little addition of 0.5 % PP and steel fibres. When the steel fibre concentration reached 2% and higher, shrinkage nearly completely disappeared [55,76]. However, when the amount of PP fibre increased to 4 %, there was a negative reaction. The same amount of PP fibre did not exhibit a commensurate decline.

**Figure 13:**
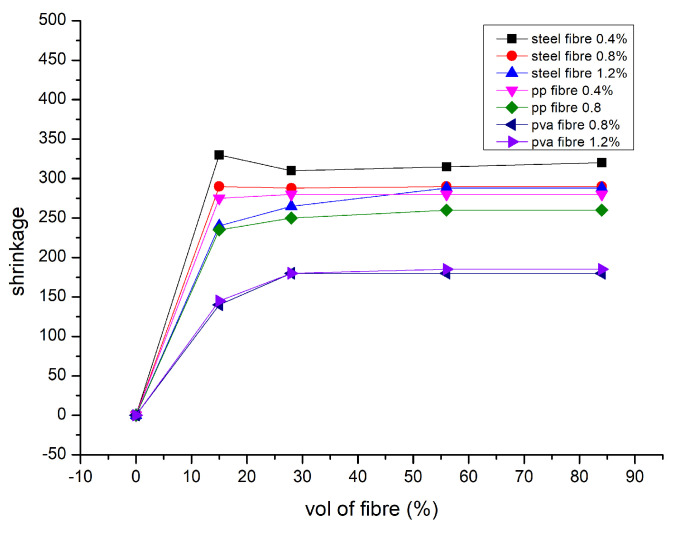
Shrinkage in micro strains [10].

The fact that geopolymer composites do not compress well when there are a lot of fibres present may help to explain this. The lower performance of PP fibres compared to steel fibre is also due to their decreased stiffness and weaker fibre–binder interaction. Because of PVA fibre’s hydrophilic qualities and higher stiffness, they exhibit comparable shrinkage performance to geopolymers reinforced with PP fibres. Shrinkage of geopolymer composites has also been observed to be (1) reduced [137], (2) increased [120], or not changed [131] when utilising longer fibres. The kind of binder and curing conditions also affect how much fibre-reinforced geopolymer composites shrink. Figure 13 represents drying shrinkage in micro strains [10].

### 9.7. Density

According to [91], the lightweight geopolymer concrete’s dry density nonlinearly reduced in foam volume. It is obvious that when dry density falls, thermal conductivity and compressive strength often decrease nonlinearly. However, it is challenging to expand the foam to minimise heat conductivity when dry density drops to about 498 kg/m^3^. The results shows poor binding results in reduced compressive strength and density of 1.60 and 1.62 g/cm^3^ respectively without reinforcement after twenty-eight days [111]. There were 1.54 and 1.48 g per cubic centimetre in the samples with flax fibre added. After just 7 days, the density of the carbon-fibre-reinforced samples was 1.67 g/cm^3^, and after 28 days, it decreased to 1.58 g/cm^3^. For various kinds of composition, density differences were not noteworthy. For this collection of samples, there is no evident link between density and mechanical properties. Density is measured under tension and compared to the yield strength of geopolymer composites and various fibres. Nearly all carbon and polymeric-based fibres are less dense than geopolymer composites, but steel and inorganic fibres have more densities. Where the composite’s lightness is a key characteristic, density variation should be considered. Regarding composite densities, the amount and specific gravity of fibres have a direct impact. When the geopolymer matrix is higher and the fibre-specific gravity is lower, the bulk density of associated geopolymer composites decreases [76]. However, when fibres with a denser composition than geopolymers are utilised, this is not always the case. As a result, when employing high-density fibre, the composite’s density may be either (1) kept at the same level, (2) lowered, or (3) raised to correspond with the plain matrix. This is because greater porosity causes the density of the composites to decrease, trapping air bubbles. As a result, the density difference is not the primary driving force. The measured density in geopolymer composites with fibre reinforcement is lower than the values suggested by the law of mixtures. A wide range of factors may influence the structure and volume of the porosity. For instance, fibres with a larger aspect ratio might have an enhanced pore volume fraction, and cellulosic fibres have a higher level of induced porosity because they expand when water is present and contract when the matrix hardens [129]. As a result, debonding manifests itself as porosity at the fibre/matrix interface [138].

### 9.8. Water Absorption

**Figure 14:**
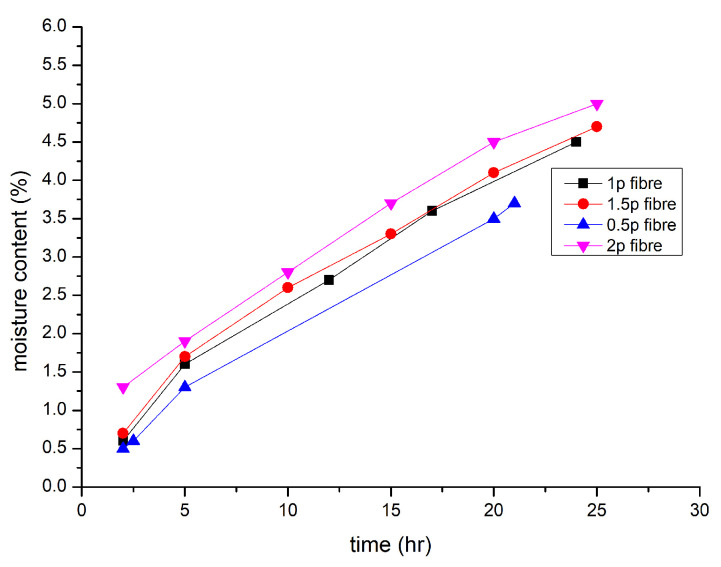
Moisture Content [10].

According to [87], better compression strength is shown by higher-grade geopolymer concrete, which implies that water absorption must be significantly reduced. Cement concrete absorbed 2.6% of the water. The geopolymeric concrete paver block of M50 grade revealed 50% less water absorption than cement concrete, which is an exciting conclusion of the finding. The results of [91] demonstrated that when the fibre length increased from 3 mm to 19 mm, the moisture content likewise climbed. Figure 14 shows the moisture content for different volumes of fibres added [10]. More moisture absorption was accomplished by increasing the fibre content in the FLGC samples from 0.5 to 2.0%. For FLGC samples with various fibre compositions, the range in moisture levels was also reduced, and surface waterproofing treatment may significantly lessen moisture absorption.

Consequently, surface waterproofing may reduce the thermal insulating capability of a material with considerable water absorption capabilities. The amount of polypropylene fibre present, which also has a negligible effect on the sample’s water absorption, directly proportionally affects how much water is absorbed by geopolymer concrete. However, a sample with a 2.5 monomer % and an increase in fibre from a little increase in the amount of water absorption is seen between 0.3 and 0.5, which may be the result of improper blending of the sample. As much as seventeen, thirteen, and fifteen vol less water is absorbed when an increase from 2 to 2.5 occurs in the sodium hydroxide to sodium silicate ratio. When more monomers were present, the amount of water absorbed was not altered considerably. Fresh concrete properties are described in Table 5 [90,92,107,116]. By comparing the amount of water absorbed, we can determine that geopolymer concrete with a monomer percentage of three reduced the quantity of water absorbed. A sample with 1% polypropylene strands had the greatest impact on cement’s ability to absorb water, causing a drop in water absorption from 4.87 to 3.78. A decrease in the size of depressions in these occurrences might be the source of the lower water absorption measurement. The porosity of the saturated example is used to determine how much water would be absorbed [56,96,139]. Figure 14 shows the moisture content [10].

### 9.9. Microstructural Property

**Figure 15:**
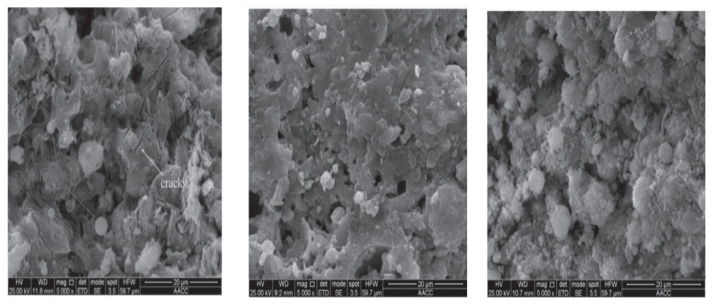
SEM of FLGC NaOH Contents, i.e., 5%, 10%, and 15% [91]. (Reprinted with permission from the publisher, Elsevier; License No. 547171129494).

Figure 15 shows the SEM of fly-ash-based lightweight geopolymer concrete (FLGC) NaOH contents, i.e., 5%, 10%, and 15% [91]. The fly ash and alkali solutions did not polymerise as well due to lower NaOH levels. There were many microcracks observed in the matrix, which could reduce the strength of the geopolymer, and it was evident that fly ash was not entirely dissolved by the alkali solutions. The compressive strength of the final products with 5% NaOH concentrations was consequently not high because of the inadequate geopolymerisation. When NaOH was adequate, which in this case study occurred at 10%, the dissolution of fly ash was hastened. Fly ash particles were not readily apparent because the majority of them were completely dissolved and covered in geopolymer gel. Early on, aluminosilicate gel precipitated due to excess hydroxide ion concentration brought on by increasing NaOH concentrations. Fly ash’s ability to leach silicon and aluminium was constrained [139]. As a result, geopolymerisation was hindered, which had an adverse effect on compressive strength. According to [87], a MK-GGBFS/CFR geopolymer was subjected to SEM examination at the age of one day. The findings indicate that the primary constituents of the geopolymer are alumina, silica, and iron. Because the raw material (Ca) concentration was low, a small quantity of calcium may be detected in GPC according to the (EDAX) spectrum energy-dispersive X-ray analysis. In the micrograph, cracked surfaces and large fissures are visible. At the age of three days, microcracks are seen in geopolymer concrete. At three days old, the geopolymer showed a substantial increase in strength due to the greater % of the mass of sodium, aluminium, silicon, and calcium. Approximately 100% of strength increased compared to the third day of age.

Sodium, alumina, and silica make up the majority of the geopolymer. The geopolymer contains sodium alumina silicate as an extra product, according to SEM and EDAX analyses. On the 28th day, the geopolymer was devoid of microcracks [140]. According to microstructural observations and statistical quantification, the radius of the unsupported (fibre-free) matrix area was larger for longer fibre lengths, and better fibre dispersion may be achieved with shorter fibre lengths. As a rather smooth steel fibre surface, the geopolymer mixture had 10% slag concentration.

**Figure 16:**
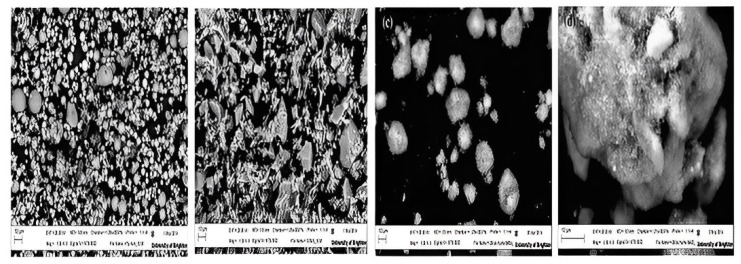
SEM images of (**a**) Fly ash, (**b**) slag, (**c**) USF, and (**d**) DSF [106]. (Reprinted with permission from the publisher, Elsevier; License No. 5471711440902).

**Figure 17:**
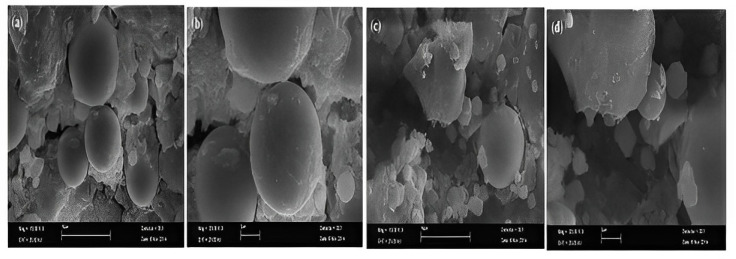
SEM analysis of 28-day geopolymer mortar [106]. (Reprinted with permission from the publisher, Elsevier; License No. 5471711440902).

On the other hand, samples with a high slag % (40%) revealed a geopolymer matrix covering the steel fibre surface. Furthermore, the observation that USF geopolymer blends result in higher degrees of hydration on the steel surface raises the possibility that the kind of SF included in the composite affects how well the fibre, i.e., steel, interacts with the matrix. Figure 16 describes fly ash, slag, USF, and DSF, as seen in SEM pictures [106]. Figure 16 shows that GGBS mostly comprises mixed-size angular particles; FA and silica fume particles both typically contain spherical and nearly spherical main particles. Larger agglomerates of silica fume particles occur in densified silica fume. By strengthening the steel fibres and matrix at the specimen contact, these hydration agents prevent pull-out failure. At the post-cracking stage, increased carrying capacity boosts the ultimate load and sample ductility. A display about the control sample B displayed about 10% DSF, and C displayed about 10% USF [106]. Figure 17 shows the SEM investigation of 28-day geopolymer mortar [106].

**Figure 18:**
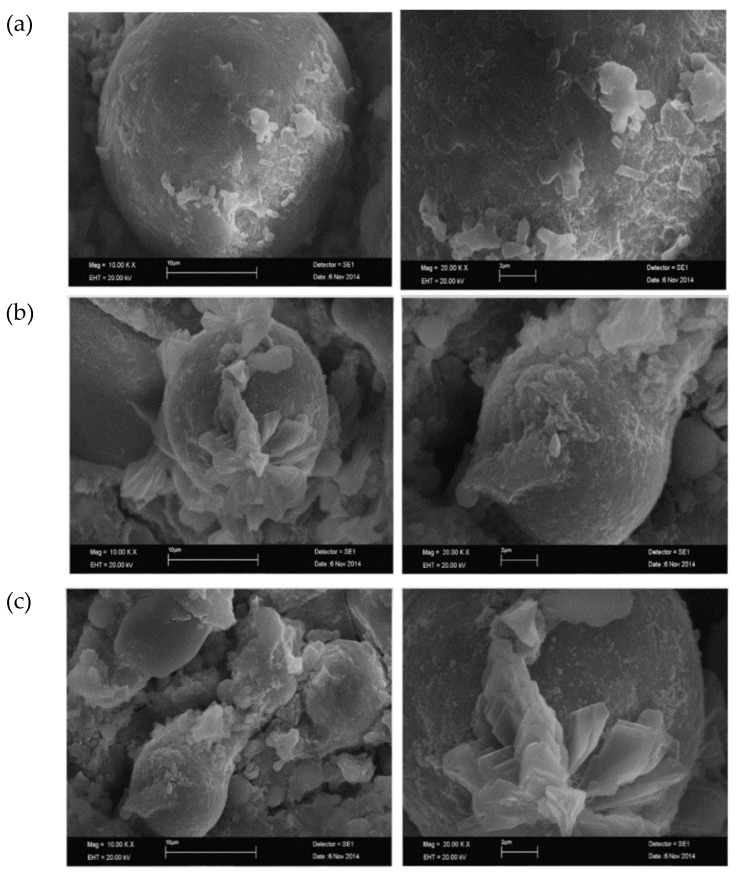
SEM images of 10% slag replacement of geopolymer mortar: (**a**) control sample; (**b**) 10% DSF; (**c**) 10% USF [106]. (Reprinted with permission from the publisher, Elsevier; License No. 5471711440902).

**Figure 19:**
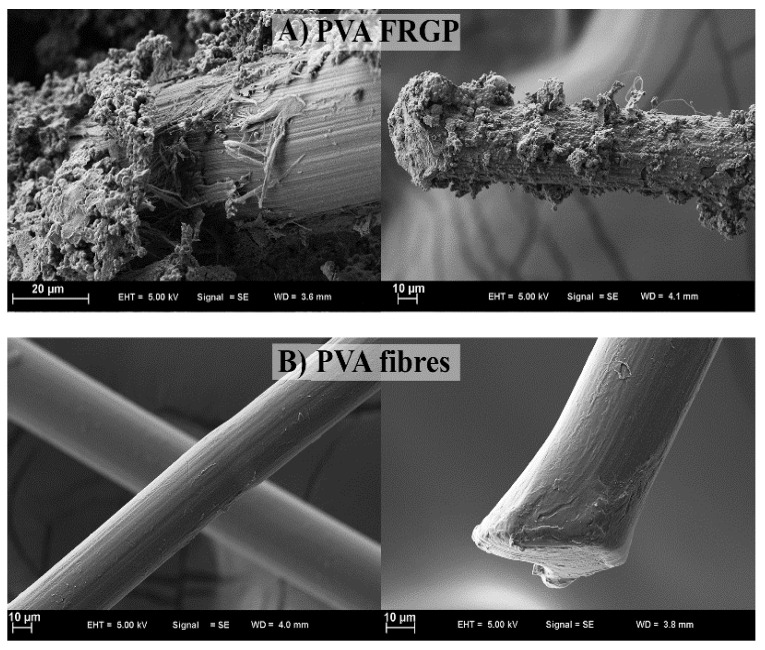
SEM micrographs showing PVA FRGP and PVA fibres [113]. (Reprinted with permission from the publisher, Elsevier; License No. 5471720272346).

In the case of the 10S mix, a large number of FA particles that were still present but only partially reacted, as well as clumped slag particles, were found. This is explained by FA’s low pozzalonic reactivity and low slag concentration when it is cured at room temperature. Higher magnification reveals distinct microstructures in the 40S combination geopolymer sample that produce a denser matrix than the 10S mixture. As a result of reactions on the surface of the particles, glassy crusts can be seen covering FA particles. A calcium alumino silicate hydrate (C-A-S-H) gel is produced as the percentage of GGBS is raised due to the mix’s higher calcium content. About 10% of the geopolymer mortar’s slag replacement is replaced in the SEM pictures shown in Figure 18. The texture of the geopolymer mortar’s hydration products with DSF was noticeably different from samples with USF and SSF. Because the large, densified silica fume particles result in lower packing and lower pozzolanic activity of the silica fume, the observed SEM picture for 10%DSF reveals no discernible difference from the control mix at ×10,000 magnification. The micrographs at higher magnification (×20,000) demonstrate that the geopolymerisation products of the mixtures containing smaller particle sizes of silica fume (for undensified (37 mm) and slurry silica (200 nm)) were composed of well-connected structures and compacted formations of hydration products were seen (Figure 19). SEM micrographs show PVA FRGP and PVA fibres [113].

**Figure 20:**
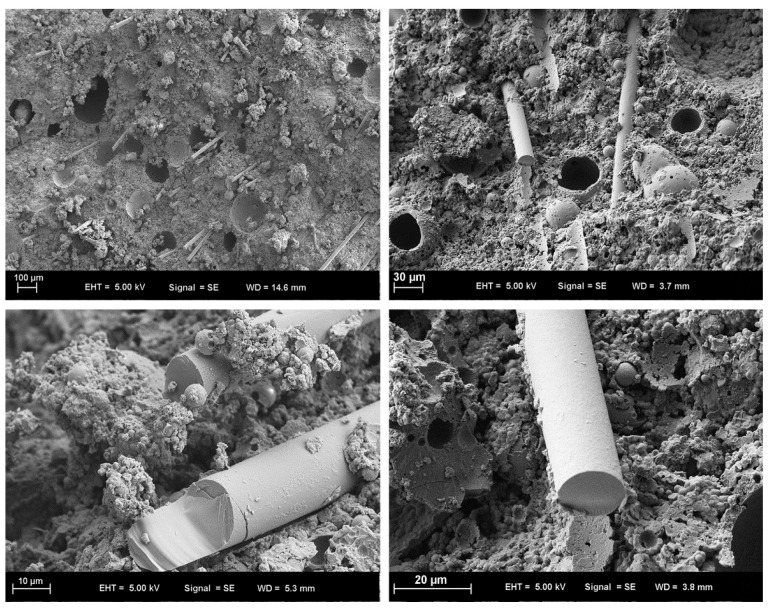
SEM micrographs showing the microstructure of basalt FRGP [113]. (Reprinted with permission from the publisher, Elsevier; License No. 5471720272346).

SEM micrographs of the basalt FRGP microstructure are shown in Figure 20 [113]. The sample’s pictures seem normal, showing that their microstructures contain a consistent distribution of fibres and no obvious big cavities. The empty space indicates that the fibre and matrix have broken their relationship, and the fibre’s shape helps explain why PP fibres function less effectively than other types of fibres. A clear correlation is seen between the flaws and poor performance of PP-fibre-reinforced geopolymers. Fly ash and alkaline solutions polymerised effectively in the control sample, according to microstructural analysis [141]. Composites made of steel and polyvinyl alcohol fibres have outstanding internal characteristics because of the fibre’s successful interfacial attachment to the geopolymeric matrix.

The performance of polypropylene fibre micrographs was inferior to that of the fibres above. The uniformity of cotton fibre dispersion in the matrix is a critical aspect in deciding how the properties of the composite behave, even though there is only a low 0.5 % cotton fibre present in the matrix. Hollow cellulose, cellulose, and hemicellulose make up around 82% of the total chemical components in lightweight concrete filler (LCF). Lignin, a chemical component also present in LCF, is thought to serve as a cellulose fibre binder and energy storage. SEM analysis indicates that more geopolymer gel is produced by the LF geopolymer composite. Coarser geopolymer gel particles and a denser microstructure are made possible by including the LF composite.

### 9.10. Crack Control

According to [105], the amount of polyethylene (PE) fibres rises with the increased frequency of numerous cracks. Additionally, there are fewer multiple fractures than hybrid composites with 1.5% PE and 0.5% SF fibres. This shows that a maximum amount of PE fibres can be used in a hybrid system with 0.5% SF fibre. According to [93], the GFRP-ROPC beams initial cracking load was higher than that of the GFRP-RGPC beams because of the lack of discrepancy in the concrete’s strength (compressive) and types. GFRP-RGPC and GFRP-ROPC beams experienced further breaking after initial breaking, the breadth of which expanded with applied force. The number of fractures in the GFRP-RGPC beams at maximum stress was almost equal to that of the GFRP-ROPC beams (number of cracks). The low GGBS concentration of SFRGC mixes led to unsatisfactory max stress and behaviour of cracks because there was insufficient bonding between the fibres, i.e., steel and low-strength GOP matrix. The inclusion of more slag and fine silica fume particles significantly increased the ability to absorb energy and post-cracking behaviour (USF). According to [24], when steel fibre activity is increased by the alkali-activated slag cement (AASC) matrix, the analysed concrete’s notch sensitivity grows as a result of the crack-arresting process. After the panels cooled to room temperature, compression testing revealed that the geopolymer concrete panels had a larger % of strength preserved than the concrete panels of OPC.

## 10. Conclusions

Based on the discussions of various studies, geopolymer concrete has been identified as a good alternative to making sustainable concrete from industrial by-products. The main drawback of geopolymer concrete is the curing method, which requires heat curing or steam curing; however, in practice, for massive construction, it is quite difficult to adopt such curing and preparation of activator solution. Compared to non-fibrous composites, adding fibres to the composite often strengthens its mechanical properties. Various fibres, such as steel fibres, glass fibres, cotton fibres, polypropylene fibres, and flax fibres, can be added to geopolymer concrete to attain some desirable strength and durability properties. The density of geopolymer concrete can be achieved by adding flax fibres to it. Fibres also enhance the modulus of elasticity and impact strength to some extent. On the other hand, adding fibres to concrete resulted in reduced workability properties due to uneven dispersion of fibres. To some extent, polypropylene fibre added to geopolymer concrete resulted in reduced water absorption and moisture content. It has been noticed that there are no specified guidelines or standards available for the mix design of geopolymer concrete, such as the mix design procedure for OPC concrete. The industry has not completely accepted geopolymer concrete yet. This is mostly due to the inability to assess information at this time on the lifespan and endurance of geopolymer concrete applications or structures. Another issue is the significant degree of instability of geopolymer concrete’s financial and environmental costs. The price of geopolymer concrete is influenced by the location of the material supply, the source of the fuel, and the modes of transportation. Depending on these three factors, geopolymer cement may cost much more than OPC concrete. According to research data, researchers can focus on the following areas in the future: the development of geopolymer concrete through room temperature curing and a simplification of the preparation of activator solution for desired molarity; the formulation of specific guidelines for mix design of geopolymer concrete made by different industrial by-products; and the influence of fibres, which can be studied by blending different types and aspect ratios in geopolymer concrete.

## Figures and Tables

**Table 1:** Chemical properties of various materials.

Ref	Material Name	SO_3_	S	CaO	SiO_2_	Al_2_O_3_	Fe_2_O_3_	MgO	Na_2_O	TiO_2_	K_2_O
[90]	Fly ash	0.10	-	1.60	62.33	21.14	7.15	2.40	0.38	-	3.37
[91]	Fly ash	-	-	1.73	55.2	26.2	3.106	1.45	0.35	-	1.879
[89]	Fly ash	-	-	0.12	54.89	15.31	21.83	2.51	-	-	-
[92]	Fly ash	-	-	1.07	55.36	27.74	9.74	-	-	3.55	-
[93]	Fly ash	0.58	-	2.65	55.2	25.3	8.34	1.56	0.58	-	1.39
[94]	Fly ash	0.73	-	2.02	54.08	26.08	6.68	2.676	0.79	-	-
[95]	Fly ash	0.38	-	1.78	50	28.25	13.5	0.89	0.32	-	0.46
[90]	GGBS	0.49	-	34.12	36.40	11.39	1.69	10.30	0.35	-	3.63
[94]	GGBS	0.18	-	35.58	40.55	12.83	1.10	5.87	0.79	-	-
[89]	GGBS	-	-	2.53	42.21	21.12	7.32	2.98	-	-	-
[90]	NS	-	-	-	99.80	-	-	-	-	-	-
[93]	OPC	2.52	-	64.39	21.10	5.24	3.10	1.10	0.23	-	0.57
[93]	SF	0.05	-	-	93.0	0.2	0.05	0.51	0.2	-	0.22
[24]	GBFS	0.66	-	43.92	31.08	13.98	3.09	1.79	-	-	-
[96]	Metakaolin	0	-	0.22	52.1	43.8	2.6	0.21	0.11	-	0.32
[97]	Metakaolin	-	-	0.08	47.6	37.7	1.6	-	-	1.7	-
[97]	Ladle slag	-	-	54.5	16.4	11.1	8.7	4.0	-	0.3	-

Note: not reported (-).

**Table 2:** Physical properties of various materials.

Ref.	Material Name	LoI	SG	Surface Area (m^2^/g)	Diameter	Length	Elongation (%)	Fineness	Colour	Aspect Ratio	Bulk Density (kg/m^3^)	Dry Density (kg/m^3^)
[87]	Metakaolin	-	1.13	-	0.024	15 mm	-	-	Greyish white	-	1744	-
	Coarse aggregate	-	2.62	Grading zone 3	-	-	14.9	2.50 (Modulus)	-	-	-	-
[90]	FA	1.58	2.29	-	-	-	-	379 (Blaine)	-	-	-	-
	GGBFS	1.64	2.79	-	-	-	-	-	-	-	-	-
	NS	<1.00	2.20	-	-	-	-	-	-	-	0.91	-
[91]	PP fibre	-	-	-	0.017	-	19	-	-	-	-	1430–1740
	Standard sand	-	-	-	0.39	-	-	-	-	-	520–540	-
	Coarse aggregate	-	-	-	5–10	-	-	-	-	-	-	-
[89]	FA	0.7	2.46	-	-	-	-	7.62	-	-	-	-
	GGBS	-	2.91	-	-	-	-	3.11	-	-	-	-
	Hook end steel fibre	-	-	-	0.75	35–60	-	-	-	45–80	-	-

Note: -, not reported.

**Table 5:** Fresh concrete properties.

Fibre Type	Slump (mm)	Compaction Factor	Vee Bee Time (s)	V-Funnel Time (s)	Ref
SF	550–850	-	-	9–25	[90]
SF	77–123	-	7.9–20.3	-	[92]
Glass	140–205	-	-	-	[107]
SF	75–128	0.79–0.92			[92]
SF	0–40	-	-	-	[116]

## Data Availability

Not applicable.
